# Predicting costs of care in heart failure patients

**DOI:** 10.1186/1472-6963-12-434

**Published:** 2012-11-30

**Authors:** David H Smith, Eric S Johnson, David K Blough, Micah L Thorp, Xiuhai Yang, Amanda F Petrik, Kathy A Crispell

**Affiliations:** 1 The Center for Health Research, Kaiser Permanente Northwest, 3800 N. Interstate Avenue, Portland, OR, 97227, USA; 2 Department of Nephrology, Kaiser Permanente Northwest, 6902 SE Lake Rd Ste 100, Portland, OR, 97267, USA; 3 Department of Pharmacy, University of Washington, Magnuson Health Sciences Building, H Wing, Dean's Office, H-364, Box 357631, Seattle, WA, 98195, USA; 4 Department of Cardiology, Kaiser Permanente Northwest, 10100 South East Sunnyside Road, Clackamas, OR, 97015, USA

## Abstract

**Background:**

Identifying heart failure patients most likely to suffer poor outcomes is an essential part of delivering interventions to those most likely to benefit. We sought a comprehensive account of heart failure events and their cumulative economic burden by examining patient characteristics that predict increased cost or poor outcomes.

**Methods:**

We collected electronic medical data from members of a large HMO who had a heart failure diagnosis and an echocardiogram from 1999–2004, and followed them for one year. We examined the role of demographics, clinical and laboratory findings, comorbid disease and whether the heart failure was incident, as well as mortality. We used regression methods appropriate for censored cost data.

**Results:**

Of the 4,696 patients, 8% were incident. Several diseases were associated with significantly higher and economically relevant cost changes, including atrial fibrillation (15% higher), coronary artery disease (14% higher), chronic lung disease (29% higher), depression (36% higher), diabetes (38% higher) and hyperlipidemia (21% higher). Some factors were associated with costs in a counterintuitive fashion (i.e. lower costs in the presence of the factor) including age, ejection fraction and anemia. But anemia and ejection fraction were also associated with a higher death rate.

**Conclusions:**

Close control of factors that are independently associated with higher cost or poor outcomes may be important for disease management. Analysis of costs in a disease like heart failure that has a high death rate underscores the need for economic methods to consider how mortality should best be considered in costing studies.

## Background

Heart failure prevalence is estimated at 1% to 2% in the US, with an annual cost (direct and indirect) of over $33 billion [1]. From 1994 to 2004 deaths from heart failure increased by 28%, while the overall death rate decreased by 2% [1]. The annual cost of heart failure to insurers has recently been estimated to be greater than $8,000 per person per year [2], with cost estimates of heart failure related admissions estimated at greater than $12,000 [3].

Recent systematic reviews have highlighted the utility of disease management programs in heart failure, with reductions in heart failure hospitalizations of 27% (95% CI, 18% to 34%) [4]. Identifying heart failure patients who are most likely to suffer poor outcomes is an essential part of delivering interventions to those most likely to benefit. To date, most clinical prediction efforts in heart failure risk stratification have been undertaken among clinical trial populations and recently hospitalized patients. These studies have sought to inform clinical decision-making, for example, about whether patients presenting to the emergency department with a heart failure exacerbation should be admitted to the hospital or can be safely discharged to home [5]. These studies have included prediction of important short-term (e.g. 30 day) outcomes in heart failure including all cause mortality, and expensive events like inpatient mortality and inpatient complications [6,7]. But we sought a more comprehensive account of heart failure's events and their cumulative economic burden among a community-based population, so we predict overall expenditures. By attaching a dollar value to units of healthcare resource use, analysts can predict one comprehensive outcome, cost. Combining utilization may be especially meaningful in a disease like heart failure where the burden is not easily described with simple endpoints; a comprehensive understanding is dependent on capturing all of the ways that outcomes appear. These estimates may also be useful for modeling the cost-effectiveness of heart failure interventions.

Our analyses predict overall cost and allow us to examine the role of demographics, clinical findings, and comorbid disease in a way that can help clinicians and those responsible for population management focus their service provision. Our study uses data on a cohort of heart failure patients with near complete capture of clinical events. Knowing how strongly factors contribute to this comprehensive reflection of heart failure’s burden may yield new insights into potential strategies for burden reduction. These data can also be useful for health service expenditure planning, and for understanding how effective treatments might change costs of care. Since we focus on patients in the community setting our study is relevant to the larger set of heart failure patients. Because others have noted that patients with incident disease may differ in important ways from those with prevalent disease [8], we also explored whether costs differ by this attribute.

## Methods

### Setting

We used data from a non-profit group-model Health Maintenance Organization (HMO) that provides fully integrated health care to approximately 480,000 individuals in the Northwestern US to conduct this retrospective cohort study. The HMO has linked electronic databases that we used to collect patient level information on health care expenditure and utilization (including inpatient and outpatient visits, laboratory results and pharmacy utilization). Our study was approved by the Research Subjects Protection Office at Kaiser Permanente Northwest in compliance with the Helsinki Declaration (http://www.wma.net/en/30publications/10policies/b3/index.html).

### Patients

Patients included in the study were HMO members 20 years and older who had an echocardiogram and a diagnosis of heart failure between 1999 and 2004; they were followed for up to one year post echocardiogram (or until April 1, 2005, death, or disenrollment from the health plan, whichever came first). The patient’s first echocardiogram, plus 30 days, served as the index date for all data collection. We allowed 30 days post-echocardiogram so that we could identify incident patients whose echocardiogram was part of their heart failure diagnostic work-up. All patients had at least one year of membership (and prescription benefit coverage) prior to their index date and had between one and three years of baseline data from which baseline covariates and heart failure diagnoses were extracted. Patients had an inpatient or outpatient diagnosis of heart failure (ICD-9 428) during the baseline period; that diagnostic code has a predictive value positive of 82% for heart failure [9], but may not be the same in our setting.

### Variables

As has been done in other retrospective studies in heart failure [10], we examined clinical findings and diagnoses that are thought to be related to heart failure. The most current baseline value (before the index date) was extracted from the HMO’s electronic medical record (including laboratory data, and inpatient or outpatient visits, except where noted). Table 1 describes how we defined clinical variables.

**Table 1:** How baseline characteristics were measured

**Characteristic**	**Data source**
**Demographic characteristics**	
Age	Membership file
Gender	Membership file
Race	Membership file and electronic medical record (EMR)
BMI	EMR (BMI; weight in kg/height in m^2^)
Smoking Status	EMR
**Clinical characteristics**	
Hypertension	ICD-9 codes 401.xx – 405.xx, or systolic >140 or diastolic >90
Hyperlipidemia	ICD 9 code 272.x, or total cholesterol >199mg/dL, or low density lipoprotein >129mg/dL
Ischemic stroke	ICD 9 codes: 433.xx, 434.xx, 436, 438.0-438.53, 438.8-438.82, 438.89, 438.9, 997.02
Transient ischemic attack	ICD 9 codes: 435.xx
Hypothyroidism	ICD 9 codes: 243, 244.xx
Chronic lung disease	ICD 9 codes: 490, 491–491.21, 491.8, 491.9, 492.xx, 493.0x, 493.1x, 493.2x, 493.9x, 494.x, 495, 495.0, 495.2-495.9, 496, 518.1, 518.2
Aortic/mitral valvular disease	ICD 9 codes: 395.xx, 424.0, 424.1, 746.3-746.6, 746.81, 394.xx, 396.xx
Depression	ICD 9 codes: 296.2x-296.6x, 296.7, 296.8, 296.80, 296.82, 296.89, 300.4, 301.12, 309.1, 311
Atrial fibrillation or flutter	ICD 9 codes: 427.3x
Diabetes	Health plan diabetes registry
Coronary artery disease/acute myocardial infarction	ICD 9 codes: 410.xx – 414.xx, excluding 414.10, 414.11, 414.19
Peripheral vascular disease	ICD 9 codes: 250.7x, 440.xx, 443.81, 443.9
Left ventricular wall thickness	echocardiogram
Ejection fraction	echocardiogram
**Laboratory characteristics**	
Renal function	Outpatient laboratory values for serum creatinine (glomerular filtration rate estimated from MDRD equation)

BMI (Body mass index), EMR (electronic medical record), ICD-9 (International Classification of Diseases, 9th revision, clinical modification), MDRD (4-variable Modification of Diet in Renal Disease equation).

### Costs of care

In the costing method we used [11], standard costs for units of medical care (ie. office visits and direct hospital service components) were identified from aggregate departmental expenditures and administrative costs; other indirect and joint costs were allocated to units of direct costs. These standard unit costs were multiplied by utilization volume to obtain total costs. The pharmaceutical prices approximated retail costs in the local market. For care provided in non-HMO facilities, costs are the amounts that the HMO paid to vendors. All costs were adjusted to reflect 2005 prices. The expenditures include nearly all the costs of acute inpatient and outpatient care (fewer than 10% of members use an out-of-plan service in any given year).

### Statistical analysis

Using cost as a dependent variable requires special attention to both its functional form (right skewness) and to censoring. To address skewness we employed a natural log transformation, allowing us to use regression models based on the assumption of normality of error terms. This assumption was checked and verified with residual plots. To deal with censoring, we used the method as outlined by Baser and colleagues [12] wherein costs are recorded as a panel (monthly) dataset and inverse proportional weighting is used to derive an unbiased estimate of costs even if we have informative censoring. Weights were derived using time-to-event methods, where the event was censoring. Monthly costs within a patient are correlated, and we investigated both population-averaged models (using general estimating equations (GEE)) and patient-specific models (using random intercepts for patients) to account for this. In the case of a linear model, these two approaches are equivalent. However, GEE does not require the assumption of compound symmetry (even as a working correlation structure) as does the random intercepts model. This assumption is not entirely appropriate for repeated measures data. For that reason, we preferred the GEE approach. We performed a Hausman test to help determine whether the weighting was necessary. In our analysis we noted there were no material differences in the random versus fixed effects models and the Hausman test showed there was no statistically significant difference between the weighted and unweighted regressions; because of this we present only the unweighted, pooled ordinary least squares (POLS) results. Standard errors have been obtained using GEE with a working correlation structure of independence. Our dataset consisted of 12 monthly observations on total cost post index date for all patients. The analysis was adjusted for month by including dummy variables for month in the regression models. Because costs are known after death, we recorded monthly costs as zero after death; $1 was added to any monthly cost of zero before taking the logarithm. The above analyses were repeated, with restriction to those patients who survived the entire 12 months of follow-up. We present the results as the average monthly costs as a relative percent change from the omitted group in the regression using the formula of van Garderen and Shah [13] to obtain the exact unbiased estimator and its variance. We used alpha = 0.05 to interpret statistical significance (i.e. 95% confidence interval excludes the null), and considered a change in cost of 10% (compared to the variable’s reference group) to be economically relevant. To further investigate the relation between cost and death (by comorbid condition) we also present a Cox proportional hazards model analysis of the risk of death. We used a complete case analysis. We used SAS (SAS version 6.12 and 8.2, Cary NC) and Stata 9.2 (College Station, Texas, USA) for all analyses.

## Results

Among 519,383 adults aged 18 years and older, we found 10,265 with a diagnosis of heart failure, and 8,291 of these had an echocardiogram. Our analysis dataset included the 4,696 (of 8,291) patients who had at least one year of health plan membership and pharmacy coverage before their echocardiogram; 381 (8% of 4,696) had new-onset (incident) heart failure. Table 2 shows the baseline characteristics for the sample, stratified by whether patients had incident or prevalent disease. Patients with prevalent heart failure were older, and had a greater comorbidity load (with the exception of aortic valvular disease) than incident patients.

**Table 2:** **Characteristics of the population at baseline** (**most recent values**, **up to 3 years prior to index date**) **by prevalent and incident cohort**

	**Prevalent(n=4315)**		**Incident(n=381)**		
**Demographics**	**n**	**(%)**	**n**	**(%)**	**Total N**
Age					
20-59 years	777	(18.01%)	98	(25.72%)	875
60-64	415	(9.62%)	28	(7.35%)	443
65-69	512	(11.87%)	39	(10.24%)	551
70-74	607	(14.07%)	60	(15.75%)	667
75-79	735	(17.03%)	57	(14.96%)	792
80-84	630	(14.60%)	59	(15.49%)	689
85 years and over	639	(14.81%)	40	(10.50%)	679
Gender					
male	2061	(47.76%)	223	(58.53%)	2284
female	2254	(52.24%)	158	(41.47%)	2412
Race					
white	4074	(94.41%)	365	(95.80%)	4539
non-white	241	(5.59%)	16	(4.20%)	257
**Clinical findings and measurements**					
BMI (kg/m^2^)					
<25	1034	(23.96%)	83	(21.78%)	1117
25-34	2331	(54.02%)	219	(57.48%)	2550
>=35	950	(22.02%)	79	(20.73%)	1029
Smoking Status					
current smoker	418	(9.69%)	38	(9.97%)	456
never or former smoker	3897	(90.31%)	343	(90.03%)	4240
Systolic blood pressure (mm/hg)					
<120	1267	(29.36%)	126	(33.07%)	1393
120-140	1637	(37.94%)	149	(39.11%)	1786
>140	1411	(32.70%)	106	(27.82%)	1517
Hemoglobin					
<11gm/dl	466	(10.80%)	42	(11.02%)	508
>= 11gm/dl	3849	(89.20%)	339	(88.98%)	4188
Renal function* (ml/min/1.73m^2^)					
>=60	2389	(55.37%)	261	(68.50%)	2650
45-60	972	(22.53%)	64	(16.80%)	1036
30-45	671	(15.55%)	35	(9.19%)	706
<30	283	(6.56%)	21	(5.51%)	304
Ejection fraction on echocardiogram(%)					
>65	353	(8.18%)	18	(4.72%)	371
51-65	2331	(54.02%)	169	(44.36%)	2500
41-50	533	(12.35%)	60	(15.75%)	593
31-40	488	(11.31%)	62	(16.27%)	550
21-30	417	(9.66%)	55	(14.44%)	472
1-20	193	(4.47%)	17	(4.46%)	210
Posterior wall thickness on echocardiogram (mm)					
<11	1763	(40.86%)	154	(40.42%)	1917
>=11	2552	(59.14%)	227	(59.58%)	2779
**Comorbid diagnoses**					
Atrial fibrillation					
yes	1603	(37.15%)	123	(32.28%)	1726
no	2712	(62.85%)	258	(67.72%)	2970
Aortic valvular disease					
yes	1354	(31.38%)	141	(37.01%)	1495
no	2961	(68.62%)	240	(62.99%)	3201
Coronary artery disease					
yes	2224	(51.54%)	178	(46.72%)	2402
no	2091	(48.46%)	203	(53.28%)	2294
Chronic lung disease					
yes	2002	(46.40%)	141	(37.01%)	2143
no	2313	(53.60%)	240	(62.99%)	2553
Depression					
yes	961	(22.27%)	81	(21.26%)	1042
no	3354	(77.73%)	300	(78.74%)	3654
Diabetes					
yes	1480	(34.30%)	102	(26.77%)	1582
no	2835	(65.70%)	279	(73.23%)	3114
Hypothyroidism					
yes	726	(16.83%)	47	(12.34%)	773
no	3589	(83.17%)	334	(87.66%)	3923
Hyperlipidemia					
yes	2010	(46.58%)	181	(47.51%)	2191
no	2305	(53.42%)	200	(52.49%)	2505
Peripheral vascular disease					
yes	703	(16.29%)	49	(12.86%)	752
no	3612	(83.71%)	332	(87.14%)	3944
Stroke					
yes	474	(10.98%)	37	(9.71%)	511
no	3841	(89.02%)	344	(90.29%)	4185
Transient ischemic attack					
yes	298	(6.91%)	23	(6.04%)	321
no	4017	(93.09%)	358	(93.96%)	4375

Table 3 shows the independent contribution (and precision estimates) of demographic, clinical and comorbid factors to total monthly cost of care. The estimates are expressed as relative percent change from the omitted group; negative percents represent lower costs compared to the omitted group. Several diseases were associated with significantly higher and economically relevant cost changes, including atrial fibrillation (15% higher), coronary artery disease (14% higher), chronic lung disease (29% higher), depression (36% higher), diabetes (38% higher) and hyperlipidemia (21% higher). With the exception of hyperlipidemia, patients with these diseases had not only higher costs but also a higher death rate.

**Table 3:** Demographic, clinical and comorbid factors contribution to cost of care

		**Adjusted%change in costs over 12 months post**-**index date**: **All Patients** (**Accounting for loss to follow**-**up**, **costs recorded as zero post death**)		**Adjusted change in costs over 12 months post**-**index date**: **Survivors** (**Includes patients who did not die**; **Accounting for loss to follow**-**up**)		**Adjusted Hazard Ratio** (**HR**) **of death over 12 months post**-**index**	
**Demographics**							
Age (years)		**estimate**	**CI**	**estimate**	**CI**	**HR**	**CI**
	20-59	81.70%	(43.8% to 119.7%)	24.62%	(4.04% to 45.20%)	0.38	(0.27 to 0.53)
	60-64	85.60%	(44.8% to 126.3%)	31.74%	(9.90% to 53.58%)	0.37	(0.25 to 0.55)
	65-69	95.40%	(55.5% to 135.3%)	38.45%	(16.91% to 59.98%)	0.38	(0.26 to 0.54)
	70-74	75.80%	(41.9% to 109.7%)	30.56%	(11.53% to 49.60%)	0.48	(0.36 to 0.64)
	75-79	69.60%	(38.4% to 100.8%)	30.10%	(12.24% to 47.96%)	0.54	(0.42 to 0.71)
	80-84	24.90%	(0.4% to 49.4%)	19.94%	(2.96% to 26.93%)	0.81	(0.64 to 1.02)
	85 years and over (reference)						
Gender							
	male (reference)						
	Female	−14.80%	(−24.0% to −5.6%)	−12.74%	(20.21% to −5.27%)	1.09	(0.92 to 1.30)
Race							
	white (reference)						
	non-white	−0.10%	(−22.1% to 21.9%)	3.48%	(−14.58% to 21.54%)	1.33	(0.90 to 1.97)
**Clinical findings and measurements**							
BMI (kg/m^2^)							
	<25	−11.00%	(−26.7% to 4.6%)	13.16%	(−2.11% to 28.43%)	1.78	(1.31 to 2.42)
	25-34	−0.40%	(−13.4% to 12.6%)	4.52%	(−6.61% to 15.66%)	1.24	(0.94 to 1.63)
	>=35 (reference)						
Smoking Status							
	current smoker	−24.80%	(−37.9% to −11.8%)	−21.58%	(−32.74% to −10.41%)	1.28	(0.96 to 1.70)
	never or former smoker (reference)						
Systolic blood pressure (mm/hg)							
	<120	−8.00%	(−20.3% to4.3%)	4.29%	(−6.38% to 14.97%)	1.41	(1.14 to 1.74)
	120-140	−10.10%	(−20.5% to 0.2%)	−2.40%	(−11.08% to 6.29%)	1.26	(1.03 to 1.55)
	>140 (reference)						
Hemoglobin							
	<11gm/dl	−20.10%	(−36.7% to −3.6%)	33.27%	(14.18% to 52.37%)	2.08	(1.70 to 2.53)
	>= 11gm/dl (reference)						
Renal function* (ml/min/1.73m^2^)							
	>=60	24.60%	(−10.1% to 59.4%)	−44.73%	(−54.85% to −34.61%)	0.35	(0.27 to 0.45)
	45-60	38.10%	(−1.6% to 77.8%)	−36.75%	(−48.68% to −24.81%)	0.40	(0.30 to 0.52)
	30-45	24.70%	(−12.8% to 62.1%)	−31.49%	(−44.97% to −18.01%)	0.57	(0.44 to 0.74)
	<30 (reference)						
Ejection fraction on echocardiogram(%)							
	>65	52.70%	(1.5% to 103.4%)	−3.00%	(−26.81% to 20.81%)	0.43	(0.29 to 0.64)
	51-65	50.70%	(7.1% to 94.3%)	−15.37%	(−33.47% to 2.74%)	0.36	(0.27 to 0.49)
	41-50	44.70%	(0.3% to 89.0%)	−14.80%	(−34.20% to 4.60%)	0.39	(0.27 to 0.56)
	31-40	47.10%	(2.1% to 92.0%)	−16.09%	(−35.00% to 2.82%)	0.37	(0.26 to 0.53)
	21-30	50.50%	(4.3% to 96.8%)	−5.38%	(−26.83% to 16.07%)	0.44	(0.31 to 0.64)
	1-20 (reference)						
Posterior wall thickness on echocardiogram (mm)							
	<11	9.70%	(−2.0% to 21.4%)	9.83%	(69.90% to 18.96%)	1.10	(0.92 to 1.30)
	>=11 (reference)						
**Comorbid diagnoses**							
Atrial fibrillation	yes	15.30%	(3.1% to 27.6%)	26.75%	(16.72% to 36.79%)	1.08	(0.91 to 1.27)
	no (reference)						
Aortic valvular disease	yes	0.40%	(−10.5% to 11.3%)	9.25%	(35.73% to 18.14%)	1.22	(1.03 to 1.44)
	no (reference)						
Coronary artery disease	yes	13.90%	(1.9% to 26.0%)	19.86%	(10.19% to 29.53%)	1.09	(0.91 to 1.31)
	no (reference)						
Chronic lung disease	yes	29.20%	(16.3% to 42.0%)	44.95%	(33.82% to 56.08%)	1.25	(1.06 to 1.47)
	no (reference)						
Depression	yes	36.20%	(18.6% to 53.8%)	64.00%	(48.85% to 79.16%)	1.31	(1.08 to 1.58)
	no (reference)						
Diabetes	yes	37.80%	(22.5% to 53.0%)	64.82%	(51.37% to 78.26%)	1.38	(1.16 to 1.65)
	no (reference)						
Hypothyroidism	yes	1.40%	(−13.1% to 15.8%)	21.20%	(9.36% to 33.05%)	1.25	(1.02 to 1.52)
	no (reference)						
Hyperlipidemia	yes	21.40%	(8.4% to 34.4%)	14.35%	(5.11% to 23.60%)	0.87	(0.73 to 1.04)
	no (reference)						
Peripheral vascular disease	yes	13.20%	(−4.0% to 30.3%)	37.31%	(23.46% to 51.16%)	1.33	(1.10 to 1.62)
	no (reference)						
Stroke	yes	−0.80%	(−19.0% to 17.4%)	19.08%	(4.99% to 33.16%)	1.37	(1.10 to 1.71)
	no (reference)						
Transient ischemic attack	yes	18.00%	(−5.4% to 41.5%)	4.44%	(−11.27% to 20.16%)	0.84	(0.63 to 1.14)
	no (reference)						

Some covariables showed significant, economically relevant cost changes in an unexpected direction (i.e. that patients with the factor had lower costs) including older age, anemia (defined as hemoglobin <11mg/dl [14]), and lower ejection fraction. But examining the hazard ratio (HR) on mortality in Table 3 reveals that in each case the patients are significantly more likely to die in the presence of these variables. For example, compared with the oldest age cohort (85 years and above) patients aged 20–59 years had a HR of 0.38 (95% CI 0.27 to 0.53); patients with anemia were twice as likely to die (HR = 2.08, 95% CI 1.70 to 2.53); and compared with the worst level of ejection fraction, patients with normal ejection fraction (51% – 65%) were less likely to die (HR = 0.36, 95% CI 0.27 to 0.49). When we restricted our cost analysis to those patients who survived the entire 12 month period we found cost changes in the expected direction for patients with anemia (33.27% higher, 95% CI 14.18% to 52.37%) and ejection fraction (for example, patients with normal ejection fraction had costs 15% lower (95% CI −33.47% to 2.74%) compared with those who had the lowest level of ejection fraction). Patients at younger ages were still found to be significantly more costly even in the survivors-only cost analysis.

We undertook an exploratory analysis (data not shown) comparing relative changes in average monthly costs by incident and prevalent patients (separately). We found that ejection fraction and coronary artery disease switched signs (e.g. from higher cost to lower cost) between incident and prevalent patients. Among prevalent patients, a normal ejection fraction (51% to 65%) was associated with higher costs (71%; 95% CI, 26% to 133%) compared with the worst ejection fraction, but the opposite held for incident patients (−54%; 95% CI, -76% to −13%). For coronary artery disease, incident patients had lower costs (−40%; 95% CI −59.2% to 11.2%), while prevalent patients had costs that were 20% higher (95% CI 7.7% to 34.5%).

## Discussion

Several diseases were associated with significantly higher and economically relevant cost changes, including atrial fibrillation, coronary artery disease, chronic lung disease, depression, diabetes and hyperlipidemia. Since the presence of these factors lead to higher resource utilization they are logical candidates for inclusion in risk stratification selection processes for care management programs and for clinical attention in these programs. Some of our findings were counterintuitive in that patients with advanced age, worse ejection fraction and anemia had lower costs. These findings are surprising, but for anemia and ejection fraction are probably best explained by the greater risk of death. Patients with worse ejection fraction and anemia were less costly because they died at a higher rate. Like the other comorbid conditions listed above, these two factors are also logical candidates for risk selection and management. Why older patients have lower costs is not clear from our analysis. One potential explanation is a survivor effect.

Our finding that patients with certain conditions have lower costs has implications for research into the burden of disease. One implication is that in a disease like heart failure where patients die at a high rate, using cost of care as an outcome cannot capture all clinically meaningful events (e.g. death), and so fails to synthesize in a way that may matter to patients, clinicians, payers and policy makers. When investigators are interested in following costs for a cohort of patients, costs after a patient dies are recorded as zero, because that patient’s health care costs are fully known (zero) after death. This practice accurately reflects the real costs of care for a cohort over time, but may not do so in a way that is meaningful for all applications. For example, since physicians obviously no longer provide care to patients following death, their interest in the cost of patient care ceases. Our work offers a descriptive solution by presenting cost and death findings in a way that allows the reader to examine them in parallel. Using a survivors-only cost analysis as an adjunct is one way to illustrate how burden of illness changes in the presence of comorbidities - without the selection bias introduced by death. But this solution is not completely satisfactory because it may not yield cost estimates that are useful for a full economic evaluation because patients who die are an important part of the cost function.

Work in other areas has shown that focus on incident versus prevalent patients is a key to differentiating patients’ risk profiles; [8] our exploratory analysis suggests that further work comparing incident and prevalent heart failure patients may usefully discriminate those at risk of high cost. Specifically, patients with incident heart failure and low ejection fractions (< 20%) had higher costs than patients with incident heart failure and higher ejection fractions, but opposite findings were observed for the full cohort of prevalent patients. The explanation for this discrepancy may lie in the relatively high procedural costs surrounding events that led to the heart failure diagnosis. For example, a patient who has an acute myocardial infarction may undergo a workup that included an echocardiogram, leading to an incident diagnosis of heart failure. Acute events, such as myocardial infarction may also be associated with other expensive interventions including coronary artery bypass surgery or angiography, thus leading to significant costs among incident patients. High cost events may be associated with more severe decompensation as well, reflecting the expense of lower ejection fractions among incident patients compared with those of higher ejection fractions. Another explanation may involve the severity of the initial diagnosis with intense early management of those with the poorest ejection fraction, followed by a higher death rate as time passes. However, these exploratory findings may also have been due to chance, as we made multiple comparisons with these groupings.

Our findings are similar to recent work by Dunlay et al. [15]. who investigated the lifetime cost of care in patients with incident heart failure. They showed, for example, that diabetes and preserved ejection fraction were associated with higher costs. However, they found that anemia was associated with 10% higher costs, while our study showed anemia to be associated with 20% lower costs. Much of this difference could be due to population selection differences, since we examined incident and prevalent patients and followed them for 12 months. We did find comparable results (anemia associated with a 33% increase in costs) when our analysis was restricted to patients who survived.

Our study was limited to a single site and to patients who were members of an HMO. But the integrated care at the HMO allowed us to track comprehensive utilization across time and to examine demographic and clinical findings for these patients. Although the cost structure in the HMO is likely not completely transferable, investigators hypothesize that the ratio of costs, like those we presented in this paper, are more likely to meaningfully compare across systems of health care [16]. Another limitation to our analysis is that due to sample size considerations we did not evaluate interaction effects, for example anemia and chronic kidney disease. In the case of economically meaningful interaction between factors, we may have over- or under-estimated the true contribution of these factors to cost of care.

We used the receipt of an echocardiogram as an index date to have a common event in a patient’s natural history of heart failure. But using the date of an echocardiogram meant that the patients were medically supervised more closely; the absolute estimates of one year costs may be inflated when compared with other points in the natural history of heart failure.

### Conclusion

In conclusion, previous studies of the costs related to heart failure have largely focused on the overall burden of disease [2,3]. Some analyses that have examined the contribution of comorbid conditions were limited by access to clinical information [17]. Our study examines the question of burden in heart failure from a different perspective and has shown that 1) cost patterns may differ for newly diagnosed and prevalent patients, and 2) that several conditions (atrial fibrillation, coronary artery disease, chronic lung disease, depression, diabetes and hyperlipidemia) contribute independently to the cost of care in heart failure. These comorbid conditions may be important targets for disease management efforts. Further research could be usefully aimed toward a better understanding of how to best report the results of economic studies in conditions with a high death rate, and toward understanding reasons for the cost differences between patients with incident and prevalent disease.

## Competing interests

This study was sponsored by Amgen through a contract to the Kaiser Permanente Northwest Center for Health Research. The contract guaranteed publication rights to the authors. Amgen had the right to review and comment on the paper, but the study design, analysis, interpretation, writing and final decisions over content rested with the investigators, not the sponsor. The manuscript contains analyses of the costs and natural history of heart failure. The work is not product-specific as no pharmaceutical agents or other medical products are compared or described. The authors declare that they have no competing interests.

## Authors’ contributions

DS, EJ, MT, DB and KC participated in the design of the study and interpretation of analyses. DB carried out the statistical analysis. XY and AP extracted data from KPNW files and participated in the interpretation of the analysis. DS and EJ drafted the manuscript, and all authors read and approved the final manuscript.

## Pre-publication history

The pre-publication history for this paper can be accessed here:

http://www.biomedcentral.com/1472-6963/12/434/prepub
